# Supplementary material for: Chromothripsis during telomere crisis is independent of NHEJ, and consistent with a replicative origin
Source: Genome Res. 2019 May;29(5):737–49. doi: 10.1101/gr.240705.118 (PMC6499312; doi:10.1101/gr.240705.118)
Supplement: Supplemental Material [file supp_gr.240705.118_Supplemental_file_1.zip › contigs/annotated_contigs/DB54/contig.3.DB54_length_642_mean_cov_5.90342679128.docx]

**DB54_length_642_mean_cov_5.90342679128**

```
GAAGGTTAAAAAGCACAGAAAAAAAGCAATGGCAAC|CCAGCCAAAAAAAAAAAAAAAAAAAAAA|T|AAAAAAAAAAAAAAAAAAATT
                                     >chr12:122300608-122300636 - E=4e-02 p=0e+00  >ch
CCATATTTTCCAAGGTAAGCAATATAAATGGGGCTCAGGAATACCCCATTAGATTAAAAAAAATCTCCTAGAGAGAGAACCCTTGAGCC
r4:13384190-13384336 - E=4e-76 p=1e-03
TACAAACTAAAGACTTTCAATATAATTTTAAAT|AAA|ACATACATGTGGCCAACAAACATGAAAAAATGCTTCAGCATTACTAATCAC
                                  >chr4:13286789-13287223 - E=2e-246
TGGGAAAGGCAAATTGAAACCACAATGAGATACCATCTCACAACAGTCAGAATGGCTGTTATTAAAAAGACAAAAAAATAACAGTGGCA

AAGTTGCAAAGAAAAGGGAACAGTTACCTACTGCTGGTGAGAATGTAAGTTAGTTTAACTACTGTGGAAAGCAGTTTGGAGATTTCTCA

AAGAATTTAAAACAGAACTACCATTTGATCCAACCATCCATCCCATTGCTGAGTATATATCCATGGAAATATAAATTGTTCTACTATAA

AGACACATGCAAATTTATATTCATCACAGCAGTATTCACAATAGCAAAGATATGGAATCAACCCAGCTGCCTATCAATAGTGCATTGGA

TAAAAAAAAATGTGGTACATATAT
```
